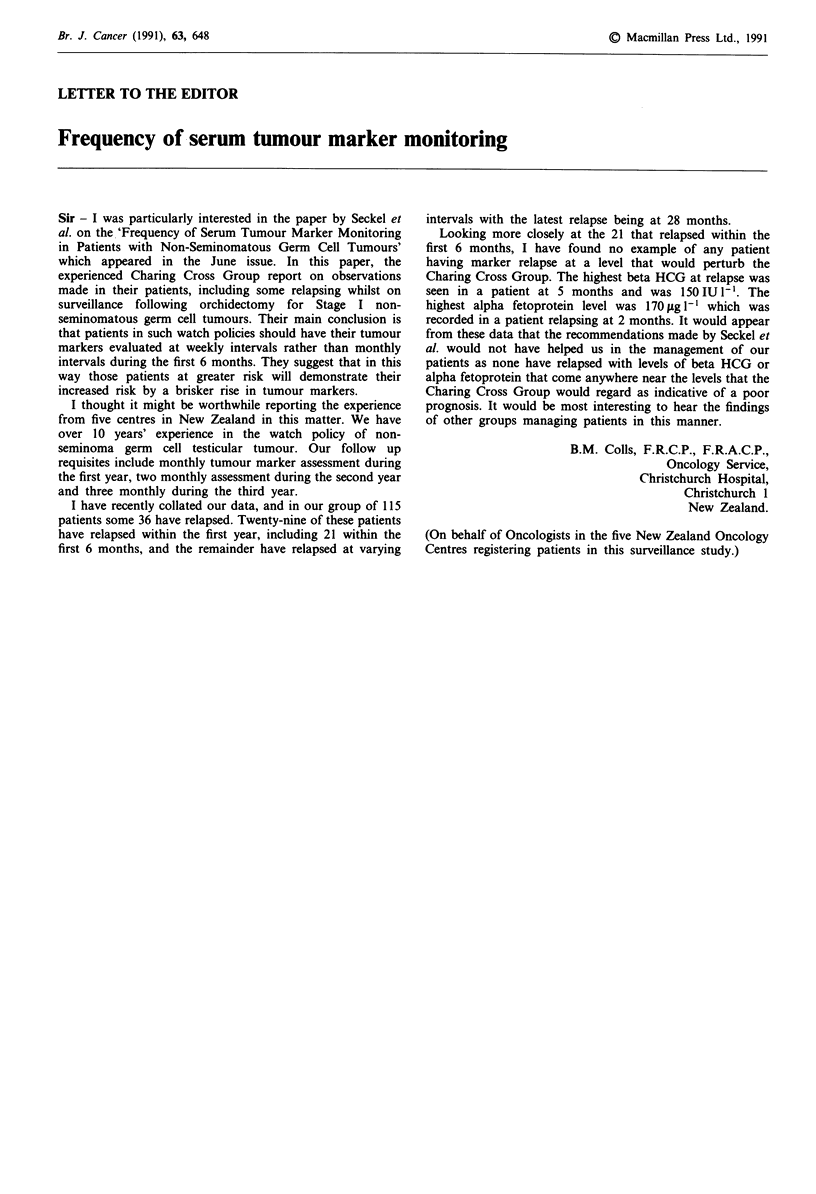# Frequency of serum tumour marker monitoring.

**DOI:** 10.1038/bjc.1991.149

**Published:** 1991-04

**Authors:** B. M. Colls


Br. J. Cancer (1991), 63, 648
© Macmillan Press Ltd., 1991

### LETTER TO THE EDITOR

### Frequency of serum tumour marker monitoring

**Sir** – I was particularly interested in the paper by Seckel *et al.* on the 'Frequency of Serum Tumour Marker Monitoring in Patients with Non-Seminomatous Germ Cell Tumours' which appeared in the June issue. In this paper, the experienced Charing Cross Group report on observations made in their patients, including some relapsing whilst on surveillance following orchidectomy for Stage I non-seminomatous germ cell tumours. Their main conclusion is that patients in such watch policies should have their tumour markers evaluated at weekly intervals rather than monthly intervals during the first 6 months. They suggest that in this way those patients at greater risk will demonstrate their increased risk by a brisker rise in tumour markers.

I thought it might be worthwhile reporting the experience from five centres in New Zealand in this matter. We have over 10 years' experience in the watch policy of non-seminoma germ cell testicular tumour. Our follow up requisites include monthly tumour marker assessment during the first year, two monthly assessment during the second year and three monthly during the third year.

I have recently collated our data, and in our group of 115 patients some 36 have relapsed. Twenty-nine of these patients have relapsed within the first year, including 21 within the first 6 months, and the remainder have relapsed at varying intervals with the latest relapse being at 28 months.

Looking more closely at the 21 that relapsed within the first 6 months, I have found no example of any patient having marker relapse at a level that would perturb the Charing Cross Group. The highest beta HCG at relapse was seen in a patient at 5 months and was 150 IU $l^{-1}$. The highest alpha fetoprotein level was 170 $\mu g\, l^{-1}$ which was recorded in a patient relapsing at 2 months. It would appear from these data that the recommendations made by Seckel *et al.* would not have helped us in the management of our patients as none have relapsed with levels of beta HCG or alpha fetoprotein that come anywhere near the levels that the Charing Cross Group would regard as indicative of a poor prognosis. It would be most interesting to hear the findings of other groups managing patients in this manner.

B.M. Colls, F.R.C.P., F.R.A.C.P.,
Oncology Service,
Christchurch Hospital,
Christchurch 1
New Zealand.

(On behalf of Oncologists in the five New Zealand Oncology Centres registering patients in this surveillance study.)